# Revisiting the Cardioprotective Effects of Acetylcholine Receptor Activation against Myocardial Ischemia/Reperfusion Injury

**DOI:** 10.3390/ijms19092466

**Published:** 2018-08-21

**Authors:** Kannaporn Intachai, Siriporn C. Chattipakorn, Nipon Chattipakorn, Krekwit Shinlapawittayatorn

**Affiliations:** 1 Cardiac Electrophysiology Research and Training Center, Faculty of Medicine, Chiang Mai University, Chiang Mai 50200, Thailand; kannapornnan@gmail.com (K.I.); scchattipakorn@gmail.com (S.C.C.); nchattip@gmail.com (N.C.); 2 Cardiac Electrophysiology Unit, Department of Physiology, Faculty of Medicine, Chiang Mai University, Chiang Mai 50200, Thailand; 3 Center of Excellence in Cardiac Electrophysiology, Chiang Mai University, Chiang Mai 50200, Thailand; 4 Department of Oral Biology and Diagnostic Science, Faculty of Dentistry, Chiang Mai University, Chiang Mai 50200, Thailand

**Keywords:** acetylcholine, α 7 nicotinic acetylcholine receptor, cardioprotection, ischemia/reperfusion injury, muscarinic acetylcholine receptor

## Abstract

Acute myocardial infarction (AMI) is the most common cause of acute myocardial injury and its most clinically significant form. The most effective treatment for AMI is to restore an adequate coronary blood flow to the ischemic myocardium as quickly as possible. However, reperfusion of an ischemic region can induce cardiomyocyte death, a phenomenon termed “myocardial ischemia/reperfusion (I/R) injury”. Disruption of cardiac parasympathetic (vagal) activity is a common hallmark of a variety of cardiovascular diseases including AMI. Experimental studies have shown that increased vagal activity exerts cardioprotective effects against myocardial I/R injury. In addition, acetylcholine (ACh), the principle cardiac vagal neurotransmitter, has been shown to replicate the cardioprotective effects of cardiac ischemic conditioning. Moreover, studies have shown that cardiomyocytes can synthesize and secrete ACh, which gives further evidence concerning the importance of the non-neuronal cholinergic signaling cascades. This suggests that the activation of ACh receptors is involved in cardioprotection against myocardial I/R injury. There are two types of ACh receptors (AChRs), namely muscarinic and nicotinic receptors (mAChRs and nAChRs, respectively). However, the effects of AChRs activation in cardioprotection during myocardial I/R are still not fully understood. In this review, we summarize the evidence suggesting the association between AChRs activation with both electrical and pharmacological interventions and the cardioprotection during myocardial I/R, as well as outline potential mechanisms underlying these cardioprotective effects.

## 1. Introduction

Despite intensive scientific efforts, acute myocardial infarction (AMI) is still the most common cause of acute myocardial injury and its most clinically significant form [1,2]. Although early restoration of blood flow to the ischemic myocardium is the most effective treatment for AMI, reperfusion per se can induce cardiomyocyte death, a phenomenon termed “myocardial ischemia/reperfusion (I/R) injury” [3]. The autonomic balance between sympathetic and parasympathetic nervous systems plays an important role in the regulation of the cardiovascular system [4]. Disruption of cardiac parasympathetic (vagal) activity is a common hallmark of a variety of cardiovascular diseases including AMI [5]. Indeed, several experimental studies have shown that increased cardiac vagal activity exerts cardioprotective effects against myocardial I/R injury [6,7,8,9,10]. Specifically, increased vagal activity by electrical stimulation can improve cardiac function in both small and large animal models in the setting of myocardial I/R injury, suggesting that activation of the cholinergic pathway may provide therapeutic benefits [11,12]. Previous study demonstrated that chronic intermittent low-level tragus stimulation (LL-TS) can attenuate cardiac remodeling in conscious dogs with healed MI [13]. Additionally, a recent clinical study demonstrated that increased vagal activity by LL-TS could reduce myocardial I/R injury in patients with ST-segment elevation myocardial infarction (STEMI) [14]. At cellular level, acetylcholine (Ach) is a neurotransmitter by which parasympathetic activity modulates both electrical and mechanical functions of the heart [15,16]. In the setting of myocardial I/R, ACh mimics the effect of myocardial ischemic conditioning, a therapeutic strategy that protects the heart from myocardial I/R injury, suggesting that activation of ACh receptors (AChRs) is involved in the cardioprotective signaling pathways [17,18,19]. In addition, previous studies have shown that cardiomyocytes can synthesize and secrete ACh, which gives further evidence concerning the importance of the non-neuronal cholinergic signaling cascades in maintaining myocardial performance during both physiologic and pathologic conditions [20,21,22]. In the heart, there are two main types of cholinergic receptors, namely muscarinic and nicotinic receptors (mAChRs and nAChRs, respectively) [18,23,24]. However, the effects of AChRs activation in cardioprotection during myocardial I/R are still not fully understood. Here, we summarize the evidence suggesting the association between AChRs activation with both electrical and pharmacological interventions and the cardioprotection during myocardial I/R, as well as outline potential mechanisms underlying these cardioprotective effects. 

## 2. Acute Myocardial Infarction (AMI) and Pathophysiologic Mechanisms of Myocardial Ischemia/Reperfusion (I/R) Injury 

AMI is one of the leading causes of death worldwide [25]. Early myocardial reperfusion with thrombolytic therapy or primary percutaneous coronary intervention (PPCI) is the most effective treatment for reducing the final myocardial infarct size and improving cardiac function [1,2]. However, the restoration of coronary blood flow to the ischemic myocardium itself can induce myocardial cell death [3]. This phenomenon is known as myocardial I/R injury, and reduces the beneficial effect of myocardial reperfusion [3]. Additionally, reperfusion therapy has been shown to induce cardiac arrhythmia, myocardial stunning and hibernation, microvascular obstruction (no re-flow phenomenon), and lethal myocardial reperfusion injury [3,26,27,28,29]. A growing number of experimental studies have identified several critical factors that act in concert to mediate the detrimental effects of myocardial I/R injury including oxidative stress [30,31], intracellular Ca^2+^ overload [17,32], the rapid restoration of physiological pH at the time of reperfusion [33], the opening of the mitochondrial permeability transition pores (MPTP) [34,35,36,37] and inflammation [38]. Unfortunately, there currently exists no clinically established therapeutic strategy for protecting the ischemic heart from lethal myocardial reperfusion injury which occurs at the time of reperfusion. Thus, novel therapeutic interventions which can be applied prior to or at the onset of the reperfusion period are required to protect the ischemic heart from this lethal injury. Growing literature has shown that the autonomic tone imbalance has a significant role in the pathophysiology and progression of several heart diseases including AMI [4]. An increased sympathetic nerve activity and a reduction of vagal cardiac tone are shown to be pathogenic in AMI patients [5]. Thus, therapeutic interventions are focused on the inhibition of the sympathetic activation [39] and the increase of the parasympathetic activity [6,7,9,10].

## 3. Parasympathetic Modulation as a Novel Strategy for Attenuating Myocardial I/R Injury 

**Figure 1:**
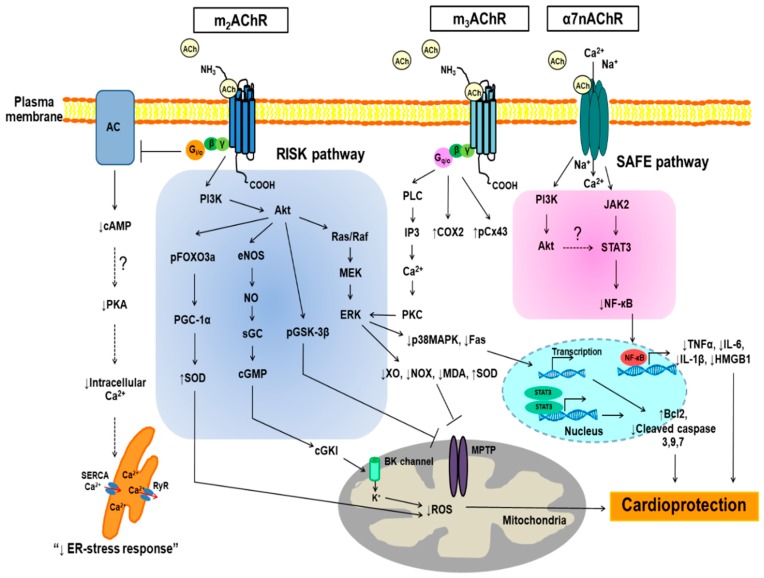
Simplified schematic representation of cardioprotective mechanisms via acetylcholine receptors in cardiomyocytes. Please note that this scheme does not entail the dimension of time. Acetylcholine confers cardioprotection against myocardial ischemia/reperfusion injury through both muscarinic and nicotinic acetylcholine receptors. The solid line indicates the known or published pathway and the dashed line represents hypothetical pathway; (⊥): proven inhibitory pathway; AC: adenylyl cyclase; ACh: acetylcholine; Akt: protein kinase B; α7nAChR: α7 nicotinic acetylcholine receptor; Bcl2: B-cell lymphoma 2; BK channel: voltage and Ca^2+^-activated potassium channel BK; cAMP: cyclic adenosine monophosphate; cGMP: cyclic guanosine monophosphate; cGKI: cGMP-dependent protein kinase type I; COX2: cyclooxygenase-2; pCx43: phosphorylated connexin 43; ER: endoplasmic reticulum; ERK: extracellular signal-regulated kinase; Fas: TNF superfamily receptor 6; HMGB1: high mobility group box 1 protein; IL-6: interleukin 6; IL-1β: interleukin 1β; IP3: inositol 1,4,5-triphosphate; JAK2: Janus kinase 2; m_2_AChR: muscarinic acetylcholine receptor type 2; m_3_AChR: muscarinic acetylcholine receptor type 3; MDA; malondialdehyde; MEK: mitogen-activated protein kinase/extracellular signal-regulated kinase kinase; MPTP: mitochondrial permeability transition pore; NF-κB: nuclear factor-kappa B; NO: nitric oxide; eNOS: endothelial nitric oxide synthase; NOX: nicotinamide adenine dinucleotide phosphate (NADPH) oxidase; pFOXO3a: phosphorylated forkhead box subfamily O3a; PGC-1α: peroxisome proliferator-activated receptor gamma coactivator-1-alpha; pGSK-3β: phospho glycogen synthase kinase 3β; PKA: protein kinase A; PI3K: phosphoinositide 3-kinase; PLC: phospholipase C; PKC: protein kinase C; p38MAPK: p38 mitogen-activated protein kinases; Ras/Raf: serine/threonine kinase; RISK: reperfusion injury savage kinase; ROS: reactive oxygen species; RyR: ryanodine receptors; SAFE: survivor activating factor enhancement; SERCA: sarco/endoplasmic reticulum Ca^2+^-ATPase; SOD: superoxide dismutase; sGC: soluble guanylyl cyclase; STAT3: signal transducers and activators of transcription 3; TNF-α: tumor necrosis factor-α; XO: Xanthine oxidase.

The heart is innervated extensively by both sympathetic and parasympathetic (vagal) nerves of the peripheral autonomic nervous system [40]. The autonomic balance of the cardiac autonomic nervous system is a crucial component in physiological and pathological responses of the cardiovascular system [4]. Disruption of parasympathetic activity is a common hallmark of a variety of cardiovascular diseases including AMI [5]. The results from preclinical studies have shown that increased vagal activity exerts cardioprotective effects against myocardial I/R injury [6,7,8,9,10]. At cellular level, ACh, a neurotransmitter of the cardiac vagus nerve, is the chemical released which acts as a stimulus for the modulation of the parasympathetic activity involved in both electrical and mechanical functions of the heart [11,12,41]. In addition, ACh has been shown to replicate the effect of cardiac ischemic conditioning (a therapeutic strategy for protecting organs or tissue against the detrimental effects of myocardial I/R injury) [42]. This suggests that the activation of AChRs is involved in cardioprotection against myocardial I/R injury [18,23,24]. A schematic representation of potential cardioprotective mechanisms mediated through AChRs in cardiomyocytes is shown in Figure 1.

## 4. The Effects of Muscarinic Receptors (mAChRs) Activation on Myocardial Infarct Size, Hemodynamic and Cardiac Function in the Setting of I/R

In AMI, a major key determinant of mortality in the affected patients is the infarct size [25]. Therefore, the primary aim of AMI treatment approaches to improve clinical outcome is to reduce the infarct size [3]. In preclinical studies of I/R injury, the activation of mAChRs has been shown to exert an infarct limiting effect (Table 1) The activation of mAChRs can be achieved by either pharmacological or direct-current electrical stimulation [10,43,44]. Several reports from ex vivo studies have demonstrated that the infarct size was significantly reduced by using the pharmacological activator of mAChRs [16,44,45]. Specifically, catestatin (CST), previously reported as a noncompetitive inhibitor of nAChRs, can also bind to m_2_AChR, thereby inhibiting ER stress-induced cell apoptosis through extracellular signal-regulated kinase (ERK1/2) and phosphoinositide 3-kinase (PI3 K)/protein kinase B (Akt) signaling pathway [44]. Moreover, activation of m_2_AChR by CST causes inhibition of adenylyl cyclase (AC) activity via α subunit (αi) of Gi, thereby reducing cAMP production [44], which may further attenuate ER-stress induced apoptosis. In addition, m_2_AChR activation increases the NOS (nitric oxide synthase/NO cGMP (nitric oxide cyclic guanosine-3′,5′-monophosphate) pathway, which also explains in part the anti-adrenergic effect of CST. Furthermore, a recent study demonstrated that activation of the cyclic guanosine monophosphate (cGMP)/cGMP-dependent protein kinase type I (cGKI) pathway through m_2_AChR activation affords cardioprotection via mitochondrial BK (BK) channels located at the inner mitochondrial membrane of cardiomyocytes [46]. In addition, previous in vivo studies demonstrated that vagus nerve stimulation (VNS) aided the rescue of an ischemic myocardium from reperfusion injury [6,7,9,47,48]. However, not all experimental studies using this therapeutic strategy have been positive. Buchholz et al. reported that continuous VNS (C-VNS) applied for 10 min before ischemia, significantly increased the infarct size in the rabbit hearts [41]. This discrepancy may be explained by differences in VNS protocols and species differences. In addition to VNS, brief periods of ischemia can also protect distant organs from I/R injury (Ripc) via the activation of the neural afferent vagus nerve by acting on mAChRs [48]. Our studies and others also demonstrated that low level VNS applied during ischemia, but not at the onset of reperfusion, significantly reduced the infarct size and ventricular dysfunction by acting through the mAChRs [6,7,48,49]. Recently, we have shown that VNS exerted cardioprotection against myocardial I/R injury predominantly through its efferent vagal fibers [9]. Although the m_2_AChR is the predominant functional mAChR subtypes in the heart, some responses of the heart to ACh may be mediated by other mAChR subtypes. Previous study demonstrated that the stimulation of m_3_AChR in the mammalian heart by an m_3_AChR agonist such as choline significantly reduced the infarct size [19,43]. In addition, the heart pretreated with choline had significantly decreased ischemia-induced arrhythmia, and reduced the number of total ventricular premature beats, reducing the duration of the ventricular tachycardia episode [43]. These findings suggest that the activation of muscarinic acetylcholine receptors (m_2_AChR and m_3_AChR) by either pharmacological or direct-current electrical stimulation, can trigger cardioprotective signaling cascades which act against I/R injury. The effects of mAChRs activation on myocardial infarct size, hemodynamic and cardiac function in the setting of ischemia and reperfusion are summarized in Table 1.

## 5. The Effects of α7 Nicotinic Acetylcholine Receptor (α7nAChR) Activation on Myocardial Infarct Size, Hemodynamic and Cardiac Function in the Setting of I/R 

Within the heart, there is evidence that cardioprotective effects are not only triggered through the activation of mAChRs but are also induced via the cholinergic anti-inflammatory pathway by the activation of α7nAChR. In rats subjected to I/R, pretreatment of vagal stimulation reduced infarct size and improved left ventricular function and a reduction in the incidence of ventricular fibrillation (VF) [12]. In addition, the application of C-VNS and I-VNS applied during regional ischemia and reperfusion significantly reduced the infarct size [50,51]. Moreover, the administration of mecamylamine (MEC) (a non-selective α7nAChR antagonist) and methyllycaconitine (MLA) (a selective α7nAChR antagonist) abrogated the protective effect of VNS, suggesting that VNS decreased infarct size through α7nAChR [50,51]. Furthermore, pretreatment with PNU-120596 (α7nAChR-selective positive allosteric modulator) significantly reduced myocardial infarct size [52]. In contrast, the α-bungarotoxin (selective α7nAChR antagonists) abolished all the protective effects of PNU-120596 on the heart [52]. Interestingly, treatment with PNU-282987 or other α7nAChR agonists at the onset of reperfusion led to significantly reduced infarct size [53,54], suggesting that α7nAChR might play an important role during myocardial reperfusion period. Moreover, a recent study reported that administration of GTS21, a selective α7nAChR agonist, significantly reduced the infarct size and improved left ventricular developed pressure (LVDP) and ±dP/dt compared with the control. The beneficial effects of GTS21 were blocked when co-administered with MLA, suggesting that GTS21 treatment decreased the infarct size and improved cardiac contractile function through the activation of α7nAChR [55]. In addition, Zhang et al. have also demonstrated that the infarct size in mice hearts pretreated with electroacupuncture at the Neiguan acupoint (PC6) was significantly reduced compared with the control [8]. Additionally, the serum cardiac troponin I was significantly decreased after electroacupuncture. Interestingly, MEC and MLA reversed the cardioprotective effect of electroacupuncture, suggesting that electroacupuncture at PC6 induced cardioprotective effects by activating α7nAChR [8]. These findings suggest that not only the activation of the mAChRs, but also the activation of α7nAChRs, by either pharmacological or direct-current electrical stimulation, can trigger cardioprotective signaling cascades which are effective against I/R injury. The effects of α7nAChR activation on myocardial infarct size, hemodynamic and cardiac function in the setting of ischemia and reperfusion are summarized in Table 2.

## 6. Anti-Apoptosis and Anti-Oxidative Stress against I/R-induced Cell Injury through mAChRs Activation 

Apoptosis and oxidative stress are key mediators underlying the pathogenesis during myocardial I/R injury [56,57,58]. Over the last few years, a growing number of studies have shown that mAChRs activation and the pertinent downstream signaling cascades exert anti-apoptotic effects and reduce oxidative stress in cases of I/R-induced cell injury [44,59,60,61,62,63]. In an in vitro study, catestatin (CST) pretreatment in neonatal cardiomyocytes led to inhibited I/R-induced cell apoptosis via the reperfusion injury salvage kinase (RISK) pathway [44]. Mechanistically, CST pretreatment decreased the level of cleaved caspase-9, -7, and -3 and Poly (ADP-ribose) polymerase (PARP), the number of apoptotic cells, and ER stress [44]. In addition, ERK and PI3K pathways have also been found to be involved in the protective effect of CST [44]. The selective m_2_AChR antagonist, AF-DX116, blocked these protections, suggesting that CST inhibited ER stress-induced cell apoptosis against hypoxia/reoxygenation (H/R) injury via m_2_AChR [44]. During the ischemic period, p38 mitogen activated protein kinase (MAPK) was activated leading to tumor necrosis factor-α (TNF-α)-induced myocardial injury [64]. In support of this finding, Li et al. demonstrated that ACh and SB203580 (p38MAPK inhibitor) treatment during ischemia decreased the level of TNF-α, cleaved caspase-3, p38MAPK and Jun-N-terminal kinase (JNK) phosphorylation and increased ERK phosphorylation in H9c2 cells. Co-treatment with atropine (a non-selective mAChR antagonist) or methoctramine (Meth) (a selective m_2_AChR antagonist) abolished the effect of ACh treatment under conditions of hypoxia, suggesting that ACh inhibits hypoxia-induced TNF-α production via MAPK phosphorylation through m_2_AChR [59].

Mitochondria are essential organelles that regulate cellular energy homeostasis and cellular function [65,66]. In the setting of myocardial I/R injury, mitochondria play an important role during the pathogenesis of cellular apoptosis [33]. Indeed, damage to mitochondria leads to an increase in the levels of reactive oxygen species (ROS), intracellular Ca^2+^ and cytochrome c in the cytosol, which then triggers cellular apoptosis [67]. Thus, the attenuation of mitochondrial dysfunction could preserve cell survival in cardiomyocytes during I/R injury. Therefore, the removal of damaged mitochondria through autophagy, a process also known as “mitophagy”, is thus critical for maintaining proper cellular functions [68]. Interestingly, it has been shown that ACh applied at the onset of reperfusion activated mitophagy through m_2_AChR [60]. In addition, Ach-mediated mitophagy has been shown to attenuate mitochondrial dysfunction in H9c2 cells following H/R [60]. Mechanistically, ACh has been shown to restore ATP content and decrease cleaved caspase-3, cytochrome c, mitochondrial ROS and mitochondrial swelling [60]. Methoctramine (a selective m_2_AChR antagonist) and m_2_AChR siRNA treatment reversed the beneficial effects of ACh, suggesting that ACh promoted cytoprotective mitophagy and was involved in the preservation of cardiac homeostasis against H/R injury via m_2_AChR [60]. In support of this it is now known that ACh acts as a mitochondrial nutrient by stimulating the transcription and protein expression of peroxisome proliferator-activated receptor co activator 1 α (PGC1α), the central factor for mitochondrial biogenesis [62]. Previous studies demonstrated that ACh reduced H/R injury through promoting mitochondrial function and ROS detoxification through the FoxO3a/PGC1α pathway [61,62]. In isolated cardiomyocytes exposed to H/R, ACh applied at the onset of reperfusion decreased cleaved caspase-3 and increased cell viability and hypercontraction [45]. Moreover, ACh inhibited mitochondrial and cytosolic ROS production against H/R injury via m_2_AChR [63]. Other functions of ACh involve the recovery of mitochondria DNA copy numbers and the diminishing of xanthine oxidase (XO) and NADPH oxidase (NOX) activity in H9c2 cells subjected to H/R [63]. However, atropine and m_2_AChR siRNA abolished the antioxidant and cardioprotective effects of ACh against H/R injury [63].

Additionally, during ischemia, both C-VNS and I-VNS provided cardioprotective effects including the reduction of mitochondria ROS and swelling in swine models [7]. These beneficial effects were abolished by atropine [7]. In the canine model, it has been shown that low levels of VNS significantly decreased the levels of myeloperoxidase (MPO), Bcl-2 associated-x protein (Bax) protein, serum and myocardial malondialdehyde (MDA) [49]. In addition, low levels of VNS also increased the levels of Bcl-2 protein, serum and myocardial superoxide dismutase (SOD) [49]. This information suggests that the activation of cardiac mAChRs, by either pharmacological or direct-current electrical stimulation, exerts both anti-apoptotic effects and anti-oxidative stress against myocardial I/R injury. In addition to m_2_AChR, previous studies have demonstrated that the stimulation of m_3_AChR in rat hearts by choline pretreatment activated anti-apoptotic B-cell lymphoma 2 (Bcl-2) protein and ERKs, increased endogenous antioxidant reserve (SOD) and reduced apoptotic mediators including FAS, p38MAPK and intracellular Ca^2+^ overload following I/R [19]. The administration of 4-DAMP, an m_3_AChR antagonist, reversed the beneficial effect of ACh, suggesting that choline produced cytoprotective effects against ischemic myocardial injury via m_3_AChR [19,43]. However, the potential therapeutic benefits of the m_3_AChR as a cardioprotective target need further investigation. The levels of anti-apoptosis and anti-oxidative stress through mAChRs activation against I/R-induced cell injury are summarized in Table 3.

## 7. Anti-Apoptosis and Anti-Oxidative Stress Against I/R-Induced Cell Injury through α7nAChRs Activation

Although the effects of mAChRs activation by ACh on the heart are well known, the effects of α7nAChRs activation by ACh remain largely unknown. Interestingly, previous studies have demonstrated that α7nAChR is localized in cardiac neurons, fibroblasts and cardiomyocytes [23]. In the brain, there is significant evidence for a protective role of α7nAChR during I/R, where it occurs via the activation of the survivor activating factor enhancement (SAFE) pathway. In an isolated perfused rat heart, GTS21 (α7nAChR agonist) administration significantly decreased ROS production and led to significantly reduced levels of JNK and p38MAPK [55]. The effect of GTS21 was blocked by MLA, the selective α7nAChR antagonist. Furthermore, in rat hearts subjected to regional I/R, PNU-120596 (α7nAChR agonist) pretreatment significantly increased SOD activities and attenuated myeloperoxidase (MPO) activities and malondialdehyde (MDA) contents [52]. The protective effect was abolished by α-bungarotoxin (BGT), the selective α7nAChR antagonist [52]. Moreover, during ischemia, VNS exhibited a significant reduction in the number of apoptotic cells [50]. The protective effect was abrogated by mecamylamine (MEC), a non-selective α7nAChR antagonist [50]. These pieces of information suggest that the activation of cardiac α7nAChRs also exert both anti-apoptotic and anti-oxidative stress effects leading to a reduction in myocardial I/R injury. The findings regarding anti-apoptosis and anti-oxidative stress as a result of α7nAChR activation against I/R-induced cell injury are summarized in Table 4.

## 8. Inflammation and the Cholinergic Anti-Inflammatory Pathway in the Setting of I/R Injury

Although the inflammatory response following AMI serves for heart repair, an excessive inflammatory response enhances the severity of myocardial I/R injury, which induces cardiac remodeling and heart failure [38,69]. Thus, novel therapeutic strategies to target the mediators in the instigation of an inflammatory response may be a potential and effective therapeutic modality in the prevention of myocardial I/R injury. Over the past few years, a growing number of studies have shown that the cholinergic anti-inflammatory pathway (CAP) can modulate various aspects of both the innate and adaptive immune response [70,71]. Interestingly, previous studies have demonstrated that ACh also exhibits an anti-inflammatory action [72,73]. The non-neuronal cholinergic system, which includes α7nAChRs, has been shown to modulate immune cell proliferation, T-helper differentiation, antigen presentation and cytokine production [74]. The α7nAChR is a ligand gated ion channel that consists of α- and β-subunits, which plays a necessary role in the regulation of cytokine release from macrophages [75]. Previous studies have shown that VNS can attenuate excessive cytokine production and inflammation [76]. Recent studies demonstrated that VNS applied 24 h before I/R events attenuated acute kidney injury and decreased plasma TNF-α level [77]. This protection by VNS was abolished in mice which had undergone a splenectomy and also in α7nAChR knockout mice [77]. VNS has also been found to attenuate hepatic I/R injury-induced liver apoptosis through the activation of α7nAChR [78]. Additionally, selective α7nAChR agonists have been shown to prevent tissue damage caused by the inflammatory process [79,80]. The α7nAChR activation may exert the anti-inflammatory effect in part via PI3K/Akt/signal transducers and activators of transcription 3 (STAT3) signaling pathway [79]. PNU-282987, an α7nAChR-selective positive allosteric modulator, exerts protective effects against cardiopulmonary bypass-induced acute lung injury and inhibits high mobility group box-1 (HMGB1) release [81]. The activation of α7nAChR also exerts protective effects against acute lung injury following I/R [81]. The activation of α7nAChR attenuated lung oxidative stress and inflammation through the suppression of the TLR4/NF-κB pathway, results in a reduction in cell apoptosis and lung injury [82]. A previous in vitro study demonstrated that the activation of α7nAChRs on Kupffer Cells (KCs) significantly reduced hepatic ischemia-reperfusion (HIR)-induced liver apoptosis by reducing ROS and H_2_O_2_ production [78]. These findings suggest that selectively targeting α7nAChRs could offer a novel therapeutic modality leading to the attenuation of I/R injury.

It has been shown that ACh can inhibit hypoxia induction of the release of HMGB1 via the activation of α7nAChR [8]. In addition, another study has reported that PNU-120596 (α7nAChR-selective positive allosteric modulator) significantly decreased the levels of serum TNFα, IL-6 and NF-κB p65 protein expression [52]. This protection was abolished by a selective α7nAChR antagonist (BGT) [52]. Moreover, postconditioning with PNU-282987 (selective α7nAChR agonist) significantly decreased serum TNFα concentration, IL-6 and HMGB1 levels [53,54]. This finding indicated that PNU-120596 pretreatment and postconditioning with PNU-282987 attenuated the systemic inflammatory response via α7nAChR [52,53,54]. In animal studies, VNS significantly reduced macrophage and polymorphonuclear neutrophil infiltration in hearts subjected to ischemia [50]. VNS also led to decreased serum TNFα, IL-6 concentration and neutrophil infiltration and increased α7nAChR protein expression [50]. These protective effects were abrogated by MEC, which is a non-selective α7nAChR antagonist [50]. More recently, it has been shown that the stimulation of the neiguan acupoint by electroacupuncture significantly attenuated the pro-inflammatory response against I/R injury via α7nAChR [8]. The sum of these findings leads towards the conclusion that VNS activates an anti-inflammatory pathway and inhibits both a systemic and local inflammatory reaction leading to the relief of myocardial I/R injury through the activation of α7nAChR. However, a recent study demonstrated that VNS applied during reperfusion reduced infarct size by activation of the α7nAChR independent of local or systemic anti-inflammatory responses [42]. Although the anti-inflammatory effects of VNS have been demonstrated in numerous studies of animal I/R model, human data are scarce. The anti-inflammatory processes mediated by α7nAChRs activation against I/R injury are summarized in Table 4.

## 9. Conclusions and Future Directions

Myocardial reperfusion injury following AMI is still a major cause of morbidity and mortality. Unfortunately, no clinically established therapeutic strategy for protecting the ischemic heart from the occurrence of lethal myocardial reperfusion injury at the time of reperfusion currently exists. Thus, novel therapeutic interventions which can be applied prior to or at the onset of the reperfusion period are required to protect the ischemic heart from lethal injury. For the past 50 years, a growing number of pre-clinical studies have shown that increased vagal activity exerts cardioprotective effects against myocardial I/R injury. Furthermore, increased vagal activity by electrical stimulation in patients with heart failure and in patients with ST-segment elevation myocardial infarction can improve cardiac function and quality of life suggesting that activation of the cholinergic pathway, through AChRs activation, may provide therapeutic benefit. Thus, good understanding of the effects and mechanisms of AChRs activation during I/R will be of great value in exploring effective targets for attenuating myocardial I/R injury in AMI patients. However, the physiological process in human is complicated. Future studies are required to address the gaps in designing clinical outcome studies and testing the proposed therapies in a complex clinical setting. The filling of these gaps will increase the opportunity to successfully attenuate myocardial I/R injury and reduce the morbidity and mortality rate in affected MI patients. Except for direct electrical VNS, future research should also focus on alternative ways to non-invasively stimulate the vagus nerve and cholinergic anti-inflammatory pathway. Moreover, discovery of selective ligands targeting the mAChRs or the α7nAChR may promote the development of novel and more efficacious therapeutic agents for attenuating myocardial I/R injury.

## Figures and Tables

**Table 1:** The effects of muscarinic acetylcholine receptor activation on myocardial infarct size, hemodynamic and cardiac function in the setting of ischemia and reperfusion.

Model	Study Protocol	Mode of Intervention	Major Finding	Interpretation	Ref.
**Ex Vivo**					
Sprague-Dawley rats	Langendorff perfusion Global ischemia: 30 min Reperfusion: 1 h	Pre-ischemia	CST 100 nM ↓ infarct size: 64.3% ↓ LVEDP ↑ dLVP, LV ±dp/dtmax -Atropine (10 nM) and AF-DX116 (100 nM) abrogated this protection.	m_2_AChR activation by CST reduces infarct size and attenuates myocardial I/R injury.	[44]
	Langendorff perfusion LAD ligation Ischemia: 35 min Reperfusion: 1 h	Pre-ischemia	IPC (3 cycle of 5 min-global ischemia/reperfusion) ↓ infarct size: 70.8% ↑ ACh: 88% -Hexamethonium (50 µM) and atropine (100 nM) blocked IPC protection.	IPC involved activation of the intrinsic cardiac nervous system, leading to release of ACh in ventricles via activation of mAChRs.	[16]
	Langendorff perfusion LAD ligation Ischemia: 35 min Reperfusion: 2 h	Onset of reperfusion	ACh 10^−7^ M ↓ infarct size: 17.6% -Ipratropium bromide (10^−11^–10^−4^ M) increased infarct size in a dose dependent manner.	ACh treatment reduces infarct size through mAChRs.	[45]
**In Vivo**					
New Zealand rabbits	LAD ligation Ischemia: 30 min Reperfusion: 3 h	Pre-ischemia	I-VNS (0.1 ms, 10 Hz, cycles of 10 s ON/50 s OFF) ↓ infarct size: 42.6% C-VNS (0.1 ms, 10 Hz) ↑ infarct size: 36% ↑ LVEDP, AP -Atropine (1.3–2.0 mg/kg) blocked the cardioprotective effects.	VNS performed intermittently antagonizes the sympathetic system and reduces the infarct size through mAChR activation.	[41]
	Global no-flow Ischemia: 30 min Reperfusion: 3 h	Pre-ischemia	rIPC (three cycles of 5 min of hindlimb ischemia and 5 min of reperfusion) ↓ infarct size: 59.8% ↑ LVDP, ↓ LVEDP -Spinal cord section abolished the reduction of infarct size. VNS (0.1 ms, 10 Hz) ↓ infarct size: 57.8% ↑ LVDP, ↓ LVEDP -Atropine (1.3–2.0 mg/kg) and vagus nerve section abolished the reduction of infarct size.	rIPC and VNS activate a neural afferent pathway and exert cardioprotection through mAChRs activation.	[48]
Wistar rats	LAD ligation Ischemia: 30 min Reperfusion: 3 h	Pre-ischemia	Choline (5 mg/kg) ↓ infarct size: 20.4% ↓ VT, VF ↑ pCx43	Choline treatment reduces infarct size and preserved pCx43 via m_3_AChR.	[43]
	LAD ligation Ischemia: 6 h	Pre-ischemia	Choline (5 mg/kg) ↓ infarct size: 33% ↓ LVEDP, LVSP ↑ ±dP/dt ↓ Arrhythmic scores, VT, VF -4-DAMP (0.5 µg/kg) abolished the protective effect of choline.	Choline treatment reduces infarct size and improves cardiac function via m_3_AChR.	[19]
FVB mice	LAD ligation Ischemia: 30 min Reperfusion: 2 h	Pre-ischemia	VNS (0.1 ms, 10 Hz) ↓ infarct size: 10% -Atropine (3–5 mg/kg) and wortmannin (1 mg/kg) reverse the reduction of infarct size.	Preischemic vagal stimulation reduces infarct size through mAChRs activation.	[42]
Domestic pigs	LAD ligation Ischemia: 1 h Reperfusion: 2 h	During ischemia	I-VNS (3.5 mA, pulse width 0.5 ms, 20 Hz, cycle of 21 s ON/30 s OFF) ↓ infarct size: 89% ↓ number of VT/VF, PVC C-VNS (3.5 mA, 0.5 ms, 20 Hz) ↓ infarct size: 60% ↓ number of PVC -Atropine (1 mg/kg) abolished the beneficial effects of VNS.	Both I-VNS and C-VNS reduce the infarct size and ventricular dysfunction through mAChRs.	[7]
	LAD ligation Ischemia: 1 h Reperfusion: 2 h	During ischemia	I-VNS (3.5 mA, pulse width 0.5 ms, 20 Hz, cycle of 21 s ON/30 s OFF) ↓ infarct size: 59% ↓ number of VT/VF, PVC ↑ pCx43 -Atropine (1 mg/kg) abolished the beneficial effects of VNS.	I-VNS reduces infarct size and ventricular fibrillation incidence through mAChRs.	[6]
Mongrel dogs	LAD ligation Ischemia: 1 h Reperfusion: 1 h	During ischemia	VNS (0.1 ms, 20 Hz) ↓ infarct size: 47.1% ↓ number of PVC, VT, VF and LF/HF ratio.	VNS reduces infarct size and ventricular arrhythmia.	[49]

AP: atrial pressure; CST: catestatin; C-VNS: continuous VNS; Cx43: connexin 43; dLVP: developed LV pressure; dP/dt: time derivatives of pressure; IPC: ischemic preconditioning; I-VNS: intermittent VNS; I/R: ischemia/reperfusion; LAD: left anterior descending; LF/HF: low frequency/high frequency; LVDP: left ventricular developed pressure; LVEDP: left ventricular end-diastolic pressure; LVSP: left ventricular systolic pressure; LV ±dp/dtmax: maximal rate of LV pressure development; mAChRs: muscarinic acetylcholine receptors; m_2_AChR: muscarinic acetylcholine receptor type 2; m_3_AChR: muscarinic acetylcholine receptor type 3; PVC: premature ventricular contraction; rIPC: remote ischemic preconditioning; VF: ventricular fibrillation; VNS: vagal nerve stimulation; VT: ventricular tachycardia; 4-DAMP: 4-diphenylacetoxy-*N*-methylpiperidine methiodide. AF-DX116: selective m_2_AChR antagonist; Atropine: non-selective mAChRs antagonist; Hexamethonium: ganglionic antagonist; Ipratropium bromide: non-selective mAChRs antagonist; Methoctramine: selective m_2_AChR antagonist; 4DAMP: m_3_AChR antagonist. ↓: decrease; ↑: increase.

**Table 2:** The effects of nicotinic acetylcholine receptor activation on myocardial infarct size, hemodynamic and cardiac function in the setting of ischemia and reperfusion.

Model	Study Protocol	Mode of Intervention	Major Finding	Interpretation	Ref.
**In Vivo**					
Sprague-Dawley rats	LAD occlusion Ischemia: 1 h Reperfusion: 2 h	Pre-ischemia	VNS (1 ms, 5 Hz) ↓ infarct size: 43% ↓ LVEDP ↑ LVSP, ±dP/dt ↓ incidence of VF: 81.6% ↓ remote vascular injury -MLA (10 mg/kg) and α7nAChR shRNA inhibited the protective effects for remote vascular injury.	VNS reduces infarct size and remote vascular protection via activating α7nAChR-mediated cholinergic pathway.	[12]
	LAD ligation Ischemia: 30 min Reperfusion: 2 h	Pre-ischemia	PNU-120596 (1 mg/kg) ↓ infarct size: 27.6% -BGT (1 µg/kg) abolished the effect of PNU-120596.	α7nAChR activation by PNU-120596 reduces infarct size.	[52]
	LAD ligation Ischemia: 30 min Reperfusion: 24 h	During ischemia	VNS (2.5 V, pulse width 0.5 ms, 8–10 Hz) ↓ infarct size: 87.7% -MEC (2.1 mg/kg) reduced the protective effect of VNS.	VNS decreases infarct size through α7nAChR.	[50]
	LAD ligation Ischemia: 30 min Reperfusion: 24 h	During ischemia	VNS (0.5 ms, 0.1–1 mA,15 Hz, cycle of 40 s ON/20 s OFF) ↓ infarct size: 38.8% -MLA (10 mg/kg) partially abolished the protective effect of VNS.	VNS decreases infarct size through α7nAChR.	[51]
	LAD ligation Ischemia: 30 min Reperfusion: 3 h	Onset of reperfusion	PNU-282987 (2.4 mg/kg) ↓ infarct size: 23%	α7nAChR activation by PNU-282987 protects against myocardial I/R injury.	[53]
	LAD ligation Ischemia: 30 min Reperfusion: 3 h	Onset of reperfusion	Combined ischemia postconditioning and PNU-282987 (2.4 mg/kg) ↓ infarct size: 45%	Combined ischemia postconditioning and α7nAChR activation by PNU-282987 protect against myocardial I/R injury.	[54]
	LAD ligation Ischemia: 30 min Reperfusion: 1 h	Onset of reperfusion	GTS21 (0.06–1.0 mg/kg) ↓ infarct size: 42% ↑ LVDP, ±dP/dt	α7nAChR activation by GTS21 at initial of reperfusion reduces infarct size and improved LV function.	[55]
C57BL6 mice	LAD ligation Ischemia: 30 min Reperfusion: 24 h	Pre-ischemia	Electroacupuncture (1 mA: 2 Hz and 100 Hz) at Neiguan acupoint (PC6) ↓ infarct size: 33.9% ↓ serum cardiac troponin 1 -MEC (1 mg/kg) and MLA (1 mg/kg) reversed the cardioprotective effect of electroacupuncture.	Electroacupuncture at Neiguan acupoint reduces infarct size through α7nAChR.	[8]
FVB mice	LAD ligation Ischemia: 30 min Reperfusion: 2 h	Onset of reperfusion	VNS (0.1 ms, 10 Hz) ↓ infarct size: 9% -MLA (5 mg/kg) and AG490 (4 mg/kg) reverse the reduction of infarct size.	Vagal stimulation during the first 10 min of reperfusion reduces infarct size through α7nAChR activation.	[42]

BGT: α-bungarotoxin; dP/dt: time derivatives of pressure; GTS21: 3-(2,4-Dimethoxybenzylidene)-anabaseine dihydrochloride or DMXB-A; I/R: ischemia/reperfusion; LAD: left anterior descending; LVEDP: left ventricular end-diastolic pressure; LVSP: left ventricular systolic pressure; LVDP: left ventricular developed pressure; MEC: mecamylamine; MLA: methyllycaconitine; nAChR: nicotinic acetylcholine receptor; PNU-120596: 1-(5-chloro-2,4-dimethoxy-phenyl)-3-(5-methyl-isoxanol-3-yl)-urea; PNU-282987: *N*-(3*R*)-1-Azabicyclo[2.2.2]oct-3-yl-4-chlorobenzamide; VNS: vagus nerve stimulation; VF: ventricular fibrillation; α7nAChR: α7 nicotinic acetylcholine receptor. AG490: JAK2 inhibitor; BGT: selective α7nAChR antagonists; GTS21: selective α7nAChR agonist; MEC: non-selective α7nAChR antagonist; MLA: selective α7nAChR antagonists; PNU-120596: α7nAChR-selective positive allosteric modulator; PNU-282987: selective α7nAChR agonist. ↓: decrease; ↑: increase.

**Table 3:** Anti-apoptotic and anti-oxidative stress effects as a result of muscarinic acetylcholine receptor activation against ischemia/reperfusion-induced cell injury.

Model	Study Protocol	Mode of Intervention	Major Finding		Interpretation	Ref.
			Anti-Apoptosis	Anti-Oxidative Stress		
**In Vitro**						
Neonatal rat cardiomyocytes	Hypoxia: 12 h Reoxygenation: 24 h	Pre-hypoxia	CST (100 nM) ↓ cleaved caspase-3, -9, and -7, PARP ↓ apoptotic cell, cAMP ↓ p-ERK1/2, pAkt -PD98059 (20 µM) and wortmannin (10 nM) blocked the CST protection on cell apoptosis. -AF-DX116 (100 nM) blocked the effect of CST.	-	m_2_AChR activation by CST activates ERK1/2 and PI3K/Akt pathways to inhibit ER stress-induced cell apoptosis.	[44]
H9c2 cells	Hypoxia: 24 h	During hypoxia	ACh (10^−6^ M) and SB203580 (10^−5^ M) ↓ TNFα, cleaved caspase-3, ↓ % cell death ↓ p-p38MAPK, p-JNK ↓ p-ERK -Atropine (10^−4^ M) and Methoctramine (10^−4^ M) abrogated the effect of ACh treatment.	-	ACh treatment inhibits hypoxia-induced TNFα production via MAPK phosphorylation through m_2_AChR.	[59]
	Hypoxia: 12 h Reoxygenation: 2 h	During hypoxia	-	ACh (10^−5^ M) ↓ mtROS, ↓ XO, NOX activity-Atropine (10^−4^ M) and m_2_AChR siRNA abolished the antioxidant and cardioprotective effect of ACh.	ACh treatment inhibits mitochondrial and cytosolic ROS production via m_2_AChR.	[63]
	Hypoxia: 8 h Reoxygenation: 4 h	Onset of reoxygenation	ACh (10^−6^ M) ↓ Cleaved caspase-3, cytC ↓ mtROS and mitochondria swelling ↑ ATP content, preserved mitochondrial membrane potential -Methoctramine (10^−6^ M) and m_2_AChR siRNA reversed the effect of ACh.	-	ACh treatment promotes cytoprotective mitophagy and preserved cardiac homeostasis via m_2_AChR.	[60]
	Hypoxia: 8 h Reoxygenation: 3 h	Onset of reoxygenation	ACh (10^−3^ M) ↓ Apoptotic cell ↑ ATP synthesis, ↑ mtDNA copy -Atropine (10^−3^ M), PGC-1α siRNA and AMPK siRNA blocked the effect of ACh on mitochondria function.		ACh treatment reduces H/R injury through promoting mitochondria biogenesis and function through AMPK/PGC-1α pathway via mAChRs.	[62]
	Hypoxia: 8 h Reoxygenation: 2 h	Onset of reoxygenation	ACh (10^−5^ M) ↓ Apoptotic cell ↑ ATP synthesis -Atropine (10^−4^ M), PGC-1α siRNA block the effect of ACh.	ACh (10^−5^ M) ↑ SOD1, SOD2 ↓ ROS, ∆Ψ_m_ -Atropine (10^−4^ M) reversed the effect of ACh. -FoxO3a siRNA blocked the effect of SOD activities.	ACh treatment reduces H/R injury through promoting mitochondria function and ROS detoxification through FoxO3a/PGC-1α pathway via mAChRs.	[61]
Adult isolated rat ventricular cardio- myocytes	Hypoxia: 4 h Reoxygenation: 2 h	Onset of reoxygenation	ACh (10^−7^ M) ↓ cleaved caspase-3 ↑ cell viability -Atropine (10^−7^ M) reversed the effect of ACh on cell apoptosis.	ACh (10^−7^ M) ↑ time of myocardial depolarization and hypercontraction	ACh treatment reduces apoptosis and oxidative stress via muscarinic receptors.	[45]
**In Vivo**						
Wistar rats	LAD ligation Ischemia: 30 min Reperfusion: 3 h	Pre-hypoxia	-	Choline (5 mg/kg) ↑ Hsp70, COX-2 ↓ dephosphorylated Cx43	Choline treatment exerts cytoprotective effect against ischemic myocardial injury via m_3_AChR.	[43]
	LAD ligation Ischemia: 6 h	Pre-hypoxia	Choline (5 mg/kg) ↓ Fas, p38MAPK, apoptotic cells ↑ ERK1, ERK2, Bcl-2 -4-DAMP reversed the effect of choline.	Choline (5 mg/kg) ↑ SOD ↓ MDA -4-DAMP abolished the protective effects of choline.	Choline treatment exerts cytoprotective effect against ischemic myocardial injury via m_3_AChR.	[19]
Domestic pigs	LAD ligation Ischemia: 1 h Reperfusion: 2 h	During hypoxia	-	C-VNS and I-VNS (3.5 mA, pulse width 0.5 ms, 20 Hz, cycle of 21 s ON/30 s OFF) ↓ mitochondria ROS production, swelling preserved mitochondrial membrane potential -Atropine (1 mg/kg) abolished the beneficial effects of VNS.	VNS decreases mitochondrial ROS production and swelling and prevents mitochondrial membrane depolarization via mAChRs.	[7]
Mongrel dogs	LAD ligation Ischemia: 1 h Reperfusion: 1 h	During hypoxia	VNS ↓ apoptotic cells, MPO, Bax protein ↑ Bcl-2 protein	VNS ↓ serum MDA, myocardial MDA ↑ serum SOD and myocardial SOD	VNS suppresses apoptosis and oxidative stress.	[49]

Akt: protein kinase B; Bax: Bcl-2 associated-x protein; Bcl-2: B cell lymphoma 2; cAMP: cyclic adenosine monophosphate; CST: catestatin; Cyt c: cytochrome c; Cx43: connexin43; ERK: extracellular signal-regulated kinase; Fas: TNF superfamily receptor 6; I/R: ischemia/reperfusion; LAD: left anterior descending; mAChRs: muscarinic acetylcholine receptors; m_2_AChR: muscarinic acetylcholine receptor type 2; m_3_AChR: muscarinic acetylcholine receptor type 3; MDA: Malondialdehyde; MEK: mitogen-activated protein kinase/extracellular signal-regulated kinase kinase; Meth: methoctramine; MPO: myeloperoxidase; NOX: nitrogen oxide; OX: xanthine oxidase; PARP: poly (ADP-ribose) polymerase; pJNK: phosphorylated Jun-N-terminal kinase; PI3K: phosphoinositide 3-kinase; p38MAPK: p38 mitogen-activated protein kinase; ROS: reactive oxygen species; SOD: superoxide dismutase; TNF-α: tumor necrosis factor α; ∆Ψ_m_: mitochondrial membrane potential; 4-DAMP: 4-diphenylacetoxy-*N*-methylpiperidine methiodide;.AF-DX116: selective m_2_AChR antagonist; Atropine: non-selective mAChRs antagonist; Ipratropium bromide: non-selective mAChRs antagonist; Methoctramine: selective m_2_AChR antagonist; PD98059: ERK inhibitor; wortmannin: PI3K/Akt inhibitor; SB203580: p38MAPK inhibitor; 4DAMP: m_3_AChR antagonist. ↓: decrease; ↑: increase.

**Table 4:** Anti-apoptosis, anti-oxidative stress and anti-inflammation through nicotinic acetylcholine receptor activation against ischemia/reperfusion injury.

Model	Study Protocol	Mode of Intervention	Major Finding			Interpretation	Ref.
			Anti- Apoptosis	Anti-Oxidative Stress	Anti- Inflammation		
**Ex Vivo**							
Sprague-Dawley rats	Global ischemia Ischemia: 30 min Reperfusion: 40 min	Pre-ischemia		GTS21 (1.6 × 10^−8^ M) ↓ ROS production maintenance of ∆Ψ -MLA (2.33 × 10^−7^ M) blocked the effect of GTS21.		GTS21 treatment reduces I/R injury by preserving mitochondrial membrane potential, maintaining intracellular ATP and reducing ROS production via α7nAChR.	[55]
**In Vivo**							
Sprague- Dawley rats	LAD ligation Ischemia: 30 min Reperfusion: 2 h	Pre-ischemia	-	PNU-120596 (1 mg/kg) ↑ SOD ↓ MDA, MPO -BGT (1 µg/kg) abolished the effect of PNU-120596.	PNU-120596 (1 mg/kg) ↓ serum TNF-α, IL-6 ↓ NF-κB p65 protein expression -BGT (1 µg/kg) abolished the effect of PNU-120596.	PNU-120596 treatment increases SOD activities, attenuated MPO activities and MDA contents in myocardium and decreased serum pro-inflammatory cytokine production via α7nAChR.	[52]
	LAD ligation Ischemia: 30 min Reperfusion: 24 h	During ischemia	VNS (2.5 V, pulse width 0.5 ms, 8–10 Hz) ↓ Apoptotic cell -MEC (2.1 mg/kg) reduced the protective effect of VNS.	-	VNS (2.5 V, pulse width 0.5 ms, 8–10 Hz) ↓ macrophage infiltration ↓ PMN infiltration -MEC (2.1 mg/kg) reduced the protective effect of VNS.	VNS decreases apoptotic cell, macrophage and PMN infiltration through nAChRs.	[50]
	LAD ligation Ischemia: 30 min Reperfusion: 3 h	Onset of reperfusion			PNU-282987 (2.4 mg/kg) ↓ serum TNFα, IL-6, HMGB1	Postconditioning with PNU-282987 attenuates systemic inflammatory response to myocardial I/R injury.	[53]
	LAD ligation Ischemia: 30 min Reperfusion: 3 h	Onset of reperfusion			PNU-282987 (2.4 mg/kg) ↓ serum TnI, TNFα and HMGB1	Combined Postconditioning with PNU-282987 and ischemia postconditioning attenuate systemic inflammatory response to myocardial I/R injury.	[54]
Mongrel dog	LAD ligation Ischemia: 1 h Reperfusion: 6 h	During ischemia	-	-	VNS (pulse width 0.5 ms, 10 Hz, 1.5–3 V) ↓ serum TNF-α ↓ serum IL-6 ↓ neutrophil infiltration ↑ α7nAChR protein	VNS activates anti-inflammatory pathway and inhibits the systemic and local inflammatory reaction.	[11]
C57BL6 mice	LAD ligation Ischemia: 30 min Reperfusion: 24 h	Pre-ischemia			Electroacupuncture (1 mA: 2 Hz and 100 Hz) at Neiguan acupoint (PC6) ↓ HMGB1 ↓ neutrophil infiltration -MEC (1 mg/kg) and MLA (1 mg/kg) reversed the cardioprotective effect of electroacupuncture.	Electroacupuncture attenuates pro-inflammatory responses and I/R injury via α7nAChR.	[8]

BGT: α-bungarotoxin; GTS21: 3-(2,4-Dimethoxybenzylidene)-anabaseine dihydrochloride or DMXB-A; HMGB1: high mobility group box 1 protein; IL-6: interleukin 6; I/R: ischemia/reperfusion; LAD: left anterior descending; MEC: mecamylamine; MDA: malondialdehyde; MLA: methyllycaconitine; MPO: myeloperoxidase; nAChR: nicotinic acetylcholine receptor; NFκB: nuclear factor-kappa B; PMN: polymorphonuclear neutrophils; PNU-120596: 1-(5-chloro-2,4-dimethoxy-phenyl)-3-(5-methyl-isoxanol-3-yl)-urea; SOD: superoxide dismutase; TNF-α: tumor necrosis factor-α; VNS: vagus nerve stimulation; α7nAChR: α7 nicotinic acetylcholine receptor. BGT: selective α7nAChR antagonist; GTS21: selective α7nAChR agonist; MEC: non-selective α7nAChR antagonist; MLA: selective α7nAChR antagonist; PNU-120596: α7nAChR-selective positive allosteric modulator. ↓: decrease; ↑: increase.

## Abbreviations

AC	Adenylyl cyclase
ACh	Acetylcholine
Akt	Protein kinase B
α7nAChR	α7 nicotinic acetylcholine receptor
Bcl2	B-cell lymphoma 2
BK channel	Voltage and Ca^2+^-activated potassium channel BK
cAMP	Cyclic adenosine monophosphate
cGMP	Cyclic guanosine monophosphate
COX2	Cyclooxygenase-2
pCx43	Phosphorylated connexin 43
ER	Endoplasmic reticulum
ERK	Extracellular signal-regulated kinase
Fas	TNF superfamily receptor 6
HMGB1	High mobility group box 1 protein
IL-6	Interleukin 6
IL-1β	Interleukin 1β
IP3	Inositol 1,4,5-triphosphate
JAK2	Janus kinase 2
m2AChR	Muscarinic acetylcholine receptor type 2
m3AChR	Muscarinic acetylcholine receptor type 3
MDA	Malondialdehyde
MEK	Mitogen-activated protein kinase/extracellular signal-regulated kinase kinase
MPTP	Mitochondrial permeability transition pore
NF-κB	Nuclear factor-kappa B
NO	Nitric oxide
eNOS	Endothelial nitric oxide synthase
NOX	Nicotinamide adenine dinucleotide phosphate (NADPH) oxidase
pFOXO3a	Phosphorylated forkhead box subfamily O3a
PGC-1α	Peroxisome proliferator-activated receptor gamma coactivator-1-alpha
pGSK-3β	Phospho glycogen synthase kinase 3 β
PKA	Protein kinase A
PI3K	Phosphoinositide 3-kinase
PLC	Phospholipase C
PKC	Protein kinase C
p38MAPK	p38 mitogen-activated protein kinases
Ras/Raf	Serine/threonine kinase
RISK	Reperfusion injury savage kinase
ROS	Reactive oxygen species
RyR	Ryanodine receptors
SAFE	Survivor activating factor enhancement
SERCA	Sarco/endoplasmic reticulum Ca^2+^-ATPase
SOD	Superoxide dismutase
sGC	Soluble guanylyl cyclase
STAT3	Signal transducers and activators of transcription 3
TNF-α	Tumor necrosis factor-α
XO	Xanthine oxidase
